# Difficulties implementing a mental health guideline: an exploratory investigation using psychological theory

**DOI:** 10.1186/1748-5908-2-8

**Published:** 2007-03-26

**Authors:** Susan Michie, Stephen Pilling, Philippa Garety, Paula Whitty, Martin P Eccles, Marie Johnston, Jemma Simmons

**Affiliations:** 1 Centre for Outcomes Research and Effectiveness, Department of Psychology, University College London, UK; 2 Department of Psychology, Institute of Psychiatry PO77, Henry Wellcome Building, De Crespigny Park, London, UK; 3 Institute of Health and Society, University of Newcastle upon Tyne, 21 Claremont Place, Newcastle upon Tyne, UK; 4 School of Psychology, Kings College, University of Aberdeen, Aberdeen, Scotland, UK

## Abstract

**Background:**

Evaluations of interventions to improve implementation of guidelines have failed to produce a clear pattern of results favouring a particular method. While implementation depends on clinicians and managers changing a variety of behaviours, psychological theories of behaviour and behaviour change are seldom used to try to understand difficulties in implementation or to develop interventions to overcome them.

**Objectives:**

This study applied psychological theory to examine explanations for difficulties in implementation. It used a theoretical framework derived from an interdisciplinary consensus exercise to code interviews across 11 theoretical domains. The focus of the study was a National Institute for Health and Clinical Excellence's Schizophrenia guideline recommendation that family intervention should be offered to the families of people with schizophrenia.

**Methods:**

Participants were recruited from community mental health teams from three United Kingdom National Health Service (NHS) Trusts; 20 members (social workers, nurses, team managers, psychologists, and psychiatrists) participated. Semi-structured interviews were audio-taped and transcribed. Interview questions were based on the theoretical domains and addressed respondents' knowledge, attitudes and opinions regarding the guideline. Two researchers independently coded the transcript segments from each interview that were related to each theoretical domain. A score of 1 indicated that the transcript segments relating to the domain did not appear to contain description of difficulties in implementation of the family therapy guidelines; similarly a score of 0.5 indicated possible difficulties and a score of 0 indicated definite difficulties.

**Results:**

Coding respondents' answers to questions related to the three domains 'beliefs about consequences,' 'social/professional role and identity,' and 'motivation' produced the three highest total scores indicating that factors relating to these domains were unlikely to constitute difficulties in implementation. 'Environmental context and resources' was the lowest scoring domain, with 'Emotion' scoring the second lowest, suggesting that these were likely to be areas for considering intervention. The two main resources identified as problems were time and training. The emotions that appeared to potentially influence the offer of family therapy were self-doubt and fear.

**Conclusion:**

This exploratory study demonstrates an approach to developing a theoretical understanding of implementation difficulties.

## Background

Evidence-based guidelines are produced in large numbers across the world to improve standards of health care and reduce inequalities in access to effective treatments. Despite widespread circulation and publicity of such guidelines, they are often not implemented effectively, with the result that there is a substantial gap between evidence and practice, and best health outcomes are not achieved [1,2]. In the Netherlands, an estimated 30–40% patients are not receiving evidence-based care [3]. In the United States, of a random sample of adults only 55% were receiving the recommended processes involved in acute, chronic and preventive healthcare, and as many as 20–25% have been found to receive unnecessary or potentially harmful care [4]. In the UK, an evaluation of 12 pieces of "tracer" guidance published by The National Institute for Health and Clinical Excellence (NICE) found variable implementation with pharmacological interventions, such as the taxanes and orlistat showing higher levels of implementation than procedures such as hearing aids, implantable cardioverter defibrillators, or laparoscopic surgical procedures [5]. A review of quality of care studies from the United Kingdom (UK), Australia and New Zealand primary care concluded that "in almost all studies the process of care did not reach the standards set out in national guidelines or set by the researchers themselves" [6].

Implementation depends on clinicians and managers changing a variety of behaviours, and there have been more than 300 evaluations of interventions to improve implementation[7] Overall, these have found modest effects but failed to produce a clear pattern of results favouring a particular method or principles to draw on in developing effective interventions [7,8]. If such interventions are to be successful, they need to be grounded in an understanding of why health professionals do, or do not change their behaviour. Understanding the causal mechanisms through which interventions lead to behaviour change can help to generalise findings from individual studies to other behaviours, populations and settings. In this way, theoretical understanding assists the development of appropriate and effective interventions. Despite the existence of a large number of psychological theories of behaviour and behaviour change, they are seldom used to try to understand implementation difficulties or to develop interventions to overcome them [9]. The few exceptions to this have not stimulated the incorporation of theory into implementation research [10-13].

For theory to be used in implementation research, it needs to be seen as relevant, accessible and useful, and researchers need to have expertise in behavioural theory. The relevance of theories of behaviour change would be more apparent if implementation of research findings were conceived in terms of health professional behaviour [14]. To make theory more accessible and useful, an interdisciplinary consensus exercise simplified and synthesised theoretical constructs relevant to implementation research into 12 domains [15]. These were: knowledge; skills; professional role and identity; beliefs about capabilities; beliefs about consequences; motivation and goals; memory, attention and decision processes; environmental context and resources; social influences; emotion; action plans and nature of the behaviour (the first 11 are influences on the behaviour that is described by the 12^th^). A theory-based implementation interview (TBII) was developed to assess the nature of implementation difficulties as a basis for developing intervention strategies [15]. This approach has been successfully used in a qualitative study of the reasons behind general practitioners' failure to fully implement guidelines for the management of coronary heart disease[16].

The current study applied this generic theory-based approach to elucidating difficulties of guideline implementation in a different health context, that of mental health. Here, examples of implementation difficulties come from a vignette study of 264 Dutch health professionals that found poor implementation of depression guidelines: 31% of all intention-to-treat decisions were not consistent with the guidelines[17]. A second example comes from the United Kingdom in relation to the care of patients with schizophrenia. Family interventions (FI) are an effective intervention [18,19]. A UK national clinical guideline recommends that "Family interventions should be available to the families of people with schizophrenia who are living with, or who are in close contact with the service user. In particular, family interventions should be offered to the families of people with schizophrenia who have recently relapsed, or who are considered at risk of relapse or have persisting symptoms" [20]. Family interventions in schizophrenia normally involve a meeting with a healthcare professional, the family, and the identified patient. The intervention, which is usually targeted at those patients at risk of relapse or with persistent symptoms, should normally consist of 10 one- to two-hour meetings over a six-month period. The intervention focuses on psycho-education about the disorder, problem solving/crisis management work, and specific interventions with the identified patient. Family interventions are the best validated psychosocial intervention for schizophrenia, with 18 good quality, randomised controlled trials consistently demonstrating a benefit across a wide range of health care systems [19]. Despite all of this, however, family therapy is an underused intervention [21]. Moreover, variation between service settings has been observed. For example, within one National Health Service Trust (an administrative structure responsible for inpatient and community mental health services, the latter delivered by multiple community mental health teams), the percentage of patients who had received family interventions across seven community teams ranged from 3% to 17% [22].

This paper describes the use of the theory-based implementation interview (TBII) to understand the difficulties in implementing the family intervention recommendation within NICE's Schizophrenia guideline in three UK NHS Mental Health Trusts, as a preliminary step to designing efforts to overcome them.

## Methods

### Setting and participants

Participants were selected from three UK NHS Mental Health Trusts, two inner-city (South and North London) Trusts serving similar areas of high psychiatric morbidity and the third covering a mixed population (including inner-city, suburban, and rural areas) in the North of England. Mental health trusts are the major providers of specialist mental health services in the English healthcare system, and their major means of service delivery are multi-disciplinary community team services known as Community Mental Health Teams (CMHTs). To gain a range of responses relevant to the national implementation of these guidelines, two CMHTs from each of the three Trusts were selected using two criteria: 1) they had begun the process of implementing, or were planning to implement the guideline, and 2) they were not known to be either particularly high or low implementers of the guideline. The team identification process was verified by discussion with the senior manager/clinician in the participating Trust who had responsibility for guideline implementation. The CMHTs identified were similar to other non-participating teams in the size, composition, work load, and general population served. One of the South London teams that was approached declined to participate due to work pressure, giving a sample of five. Participants were recruited from the key professional groups responsible for implementing the guidelines: social workers, nurses, psychiatrists, psychologists, and team managers.

### Procedure

The research was conducted in 2005. Invitation letters, study information sheets, and consent forms were sent to team managers to distribute to their team members. Twenty members of the participating mental health teams agreed to be interviewed (Table 1), representing about 20% of the overall sample. The interviews were structured by the TBII [16], with questions covering 11 theoretical domains. Areas of questioning covered: knowledge; skills; social/professional role and identity; beliefs about capabilities; beliefs about consequences; motivation and goals; memory, attention and decision processes; environmental context and resources; social influences; emotion; action planning. An examples of the style of question was, for beliefs about capabilities "Is (following the guideline recommendation) easy or difficult to do? What problems have you encountered? What would help you to overcome these problems?". Piloting produced few changes; ' [see Additional file 1]' for the full version. Interviews were conducted by two psychology graduates in participants' offices and were 30 to 60 minutes in duration. In order to ensure a shared understanding of the set of behaviours referred to in the guideline, at the beginning of the interview participants were asked if they had heard about family interventions as described in the guideline. If they had, they were asked to explain their understanding of it; if not, they were shown the relevant guideline text. Although some participants referred to family interventions as family therapy, it was clear that they meant that they were working with families rather than conducting formal therapy. The interviews then followed the structure of the TBII. The interviews included dialogue with clarifications requested by both interviewer and interviewee, as well as supplementary questions used if interviewees said little in response to the first question. Interviews were audio-taped and transcribed.

**Table 1:** Number of participants according to professional group and NHS Trust.

	North London	North England	South London
Social worker	2	3	1
Nurse	2	1	2
Team Manager	3	0	1
Psychologist	3	0	0
Psychiatrist	1	0	1

### Ethics

Ethics approval was granted from the Local Research Ethics Committees covering each of the three participating NHS Trusts.

### Transcript analysis

Interviewees' responses were reviewed for their conceptual relevance to each domain, and statements judged to be relevant to one or more of the domains were selected for scoring. For each participant, the total transcribed text relevant to each domain was scored 1, 0.5, or 0, depending on whether there was good, partial or no evidence that the response text related to the domain indicated a likelihood of successful implementation of the recommendation. Scores were assigned on the basis of a global impression of all the statements relevant to each domain. For example, if a rater judged that the text offered evidence that a respondent felt that he/she had control over implementing the recommendation, the rater assigned a score of 1 for the domain of "beliefs about capabilities;" if there was no evidence for this or evidence of a perceived lack of control, the rater assigned a score of 0; partial or equivocal evidence resulted in a score of 0.5. Therefore, the lower the score for a domain, the greater the indication that it was a domain that might explain poor implementation of the guideline recommendation. Total implementation scores for Trusts and professional groups were calculated as the ratio of the total score to the maximum score possible (number of individuals multiplied by the number of domains).

### Coding reliability

Two psychologists (SM and JS) with experience in mental health independently coded each interview. SM, who has considerable experience in transcript coding, trained JS in using the coding criteria with a set of transcripts of interviews about a different recommendation. For the study transcripts, their inter-rater agreement was 81%, with an overall kappa of 0.72. Two kappa scores were low. For consequences, it was 0.44 despite 90% agreement. This is explained by the use of only two coding categories for this domain (there were no instances of evidence of association with implementation). Since the kappa statistics is a chance-corrected measure of agreement, only two categories produce higher chance agreement, and thus a lower kappa despite 90% raw agreement. For emotion, it was 0.37; responses showed that this domain was ambiguous, with many interviewees interpreting the question as referring to emotion experienced in the intervention, rather than emotion influencing implementation of the intervention. The results in relation to this domain should therefore be treated with caution. For the discrepant 41 (out of 220) scores, consensus was reached by discussion.

## Results

The number and profession of participants across the Trusts are shown in Table 1.

### (1) Variability across profession and NHS Trust

As shown in Table 2, there was variation in overall scores across professional groups, with highest scores among nurses (56%), then social workers (47%), psychiatrists (41%), psychologists (30%), and lowest scores among team managers (18%). There also was variation across the three NHS Trusts: 46%, 57% and 63%. However, the wide confidence intervals shown in Table 2 mean that differences between the point estimates may not, in this sample, represent true differences, but the play of chance.

**Table 2:** Overall implementation scores by profession and Trust.

PROFESSION	Total/maximum possible	Percentage (95% confidence interval)
Nurse (n = 5)	31/55	56% (43 – 69)
Social Worker (n = 6)	31/66	47% (35 – 59)
Psychiatrist (n = 2)	9/22	41% (23 – 61)
Psychologist (n = 3)	10/33	30% (17 – 47)
Team Manager (n = 4)	8/44	18% (10 – 32)
TRUST		
North London (n = 11)	76.5/121	63% (54 – 71)
North England (n = 4)	31.5/55	57% (45 – 70)
South London (n = 5)	20.5/44	46% (32 – 60)

### (2) Implementation domains for total sample

Table 3 shows the numbers of participants (by professional group and NHS Trust) identifying each theory-based domain as a potential explanation for implementation difficulties. The three showing the highest total scores were 'beliefs about consequences,' 'social/professional role and identity,' and 'motivation' (19, 16.5 and 16.5 out of 20). This suggests that, in general, mental health team members thought that family interventions were likely to result in positive consequences, and that providing them was compatible with their perceptions of their role and identity, and that they were motivated to provide it.

**Table 3:** Number of participants (out of 20) identifying 'good' or 'partial' evidence for the explanatory potential of each domain, total scores for each domain and scores by geographical area

Domain	Knowledge			Skills			Professional role			Capabilities			Consequences			Motivation			Memory and attention			Environmental resources			Social influences			Emotion			Action plans		
	X	?		X	?		X	?		X	?		X	?		X	?		X	?		X	?		X	?		X	?		X	?	
Profession

Social Worker N = 6	4	1	1	2	1	3	0	1	5	3	2	1	0	0	6	0	3	3	1	2	3	5	1	0	3	1	2	3	0	3	4	0	2
Nurse N = 5	2	2	1	1	2	2	1	3	1	2	1	2	0	1	4	0	2	3	1	3	1	5	0	0	4	0	1	5	0	0	2	1	2
Psychologist N = 3	1	0	2	0	0	3	0	1	2	1	1	1	0	1	2	0	0	3	0	1	2	2	0	1	2	1	0	1	1	1	0	1	2
Psychiatrist N = 2	0	0	2	1	0	1	0	0	2	0	1	1	0	0	2	0	2	0	1	1	0	1	0	1	1	1	0	1	1	0	1	0	1
Team Manager N = 4	1	0	3	1	0	3	0	0	4	1	0	3	0	0	4	0	0	4	0	0	4	3	0	1	0	1	3	1	1	2	0	0	4

**Total = 20**	**10.5**			**13.5**			**16.5**			**10.5**			**19**			**16.5**			**13.5**			**3.5**			**8**			**7.5**			**12**		

**Kappa**	**0.77**			**0.67**			**0.88**			**0.70**			**0.44**			**0.56**			**0.53**			**0.69**			**0.68**			**0.37**			**0.82**		

Domain	Knowledge			Skills			Professional role			Capabilities			Consequences			Motivation			Memory and attention			Environmental resources			Social influences			Emotion			Action plans		

	X	?		X	?		X	?		X	?		X	?		X	?		X	?		X	?		X	?		X	?		X	?	
Trust

North England N = 5	2	1	2	1	1	3	0	1	4	1	2	2	0	0	5	0	2	3	1	2	2	4	1	0	3	1	1	3	0	2	2	1	2
North London N = 11	5	0	6	2	0	9	1	1	9	5	1	5	0	2	9	0	4	7	1	4	6	8	0	3	4	2	5	5	3	3	4	0	7
South London N = 4	1	2	1	2	2	0	0	3	1	1	2	1	0	0	4	0	1	3	1	1	2	4	0	0	3	1	0	3	0	1	1	1	2

Key: X (score of 0) = no evidence of the domain being relevant to the implementation of the recommendation; ? (score of 0.5) = partial evidence of association with implementation:  (score of 1) good evidence of association with implementation.

Examples of positive consequences were:

"Anything that is good for carers is going to be good for the whole system and the patient." (Social Worker, North England)

"You're going to increase a more knowledgeable, supportive environment for service users and their carers." (Nurse, North London)

And about 'social/professional role and identity,'

"I think we have a professional responsibility to, you know, utilise those methods." (Team Manager, South London)

At the other end of the scale, 'Environmental context and resources' was the lowest scoring domain (3.5 out of 20), with 'Emotion' scoring the second lowest (7.5), suggesting these to be likely reasons for non-implementation of the guideline, and areas for considering intervention.

The two main "resources" identified as problems were 'time' and 'supervision and training,' a perception that was shared across profession and Trust. These problems were presented, without specific probing.

Examples of Time comments were:

"If that's [lack of time] not taken into account on your case load, then you dig your heels in and say I just can't do this. Either that, or you run yourself into the ground and everybody leaves, cos they get burnt out and fed up" (Nurse, North England).

"Time and pressure involved. I mean it's much easier for me cos I can control my case load, but lots of other members of the team can't." (Psychologist, North London) ...

"If you've 45 on your case load and you're running around, and people get... the more people are pressed the more people are overworked, you know. The standards go down to the minimum..." (Social Worker, North England)

Examples of comments relevant to Supervision and Training were:

"I think they're [supervision and training] the biggest two." (Nurse, North England)

"We've got a basic problem of, you know, people that aren't trained in the way that the NICE guidelines would suggest." (Team manager, South London)

"Experience with supervision is hard to come by. Not every team has a psychologist, not every team has people that are trained and feeling competent in family work, and I think that's the big issue. Knowing what you're talking about." (Nurse, North England).

"There's an expectation around [that] everyone in the service team should work to the psychological models, and I don't think that many people feel that they're trained to do that." (Psychologist, North London)

The emotions that appeared to potentially influence the offer of family interventions were self-doubt and fear:

"... if you're working with people with a history of violence or a propensity to be violent, then you're always going to feel, maybe not scared, but aware. Well, maybe scared is the right word." (Nurse, North London)

## Discussion

This study applied a theoretical framework of behaviour change to help understand the factors influencing the implementation of clinical guidelines within a health service setting. The results show clear differences across theoretical domains capturing different types of factors. The finding that the domain of environmental context and resources was most highly associated with implementation difficulties is consistent with findings from other, non-theory-based studies. A six centre European study of implementing family interventions for people with schizophrenia reported that work overload, lack of time, and organisational difficulties in the service were impediments to implementation [21]. Our findings also suggest differences in implementation challenges across different professional groups, with fewer implementation difficulties among team managers than among the nurses and social workers who are more directly involved in making therapeutic decisions and delivering the service. In this study, the sample sizes from a small number of teams in the different Trusts are too small to draw any conclusions about differences between Trusts, and, in general, similar problems were reported across Trusts.

As well as identifying potential difficulties that stand in the way of successful implementation, this approach points to possible strategies to address the difficulties. For example, the differences between professional groups raise the possibility that an effective implementation strategy might be one which focused on the provision of more effective support and supervision for direct care staff, rather than one that concentrated solely on improving clinical skills (a high scoring domain). An alternative approach which also addresses the identified problems (in emotion, social influence, and resources) might be one which suggested re-structuring of the team where only a small number of designated staff members might routinely be expected to provide family interventions. This study points to a possible refinement of the advice currently provided to healthcare providers by organisations such as NICE [23,24]. Such advice stresses the structural changes necessary to support implementation at the organisational level or strategies to change individual behaviour, but perhaps does not give sufficient consideration to changes at the level of the organisation of the multi-disciplinary team.

This is a small study, using a simple coding scheme that is not without its problems. For example, a non-response in a particular domain may suggest an implementation difficulty; however, this is an inference and there may be other explanations for non-response. Therefore, the study has more value in demonstrating an approach to assessing and understanding implementation difficulties using a theoretical framework, than in providing definitive answers in this particular context. The advantage of using this theoretical approach over atheoretical approaches is two-fold. First, the assessment of implementation difficulties is comprehensive and covers all the areas that, based on relevant theory, are known to predict behaviour or bring about changes in behaviour. Second, understanding behaviour theoretically has implications for the nature and targeting of interventions that are likely to be effective. Work has begun to link theoretical domains with techniques of behaviour change – and to use the domains in developing interventions to increase implementation [25]. An example is the development and evaluation of an intervention strategy for general physicians' management of lower back pain, on the basis of the identified theoretical domains [26,27].

Further research is required to validate and refine the theoretical framework and the coding procedure employed here. Larger scale studies also are required for replication. This could also lead to developing an assessment tool appropriate for surveying larger numbers, such as a postal or web-based questionnaires rather than a personal interview, to measure the domains. Postal questionnaires have been successfully used in relation to identifying barriers and facilitators of implementation [28]. Such a questionnaire may also serve as an outcome measure for intervention evaluation.

In moving from a theory-based assessment of implementation difficulties to intervening, we need to identify relevant theories and intervention techniques. For example, if "beliefs in capabilities" is identified as a problematic domain, techniques for building self-efficacy, as outlined by Bandura and Social Cognitive Theory would be appropriate [29]. If "action planning" is identified as a problematic domain, Self-regulation Theory may provide ideas for helpful techniques, e.g., goal setting, monitoring, and implementation intentions [30]. A pilot study used a consensus method to identify relevant techniques based on the theoretical domains described above [25]. The linking of theories explaining behaviour change, or lack of behaviour change, to techniques of intervention is a further area of research needed to develop both theoretical understanding and effective interventions.

## Conclusion

This exploratory study demonstrated a method of identifying implementation difficulties using an interview based on psychological theory. Its use includes comparing implementation difficulties across settings and staff groups, and identifying areas for intervention. The theoretical base provides a systematic method for moving from diagnosis to intervention technique.

## Competing interests

SP received funding from NICE for the development of clinical practice guidelines. PW is currently seconded part-time to the Healthcare Commission, leading on Clinical Effectiveness, which includes monitoring the implementation of NICE guidance. The other authors declare they have no competing interests.

## Authors' contributions

SM was responsible for the research idea and project management, and contributed to interview design and data analysis. She wrote the first draft of the paper and subsequent re-drafts. All authors contributed to the development of the research objectives and methods, and to the writing of the paper. SP, PG and PW were involved in supporting data collection. JS conducted the interviews, coded transcripts, and helped analyse data. All authors read and approved the final research protocol and manuscript.

## Supplementary Material

Additional file 1 Interview schedule. The questions asked in the interview. Click here for file
