# Supplementary material for: Difficulties implementing a mental health guideline: an exploratory investigation using psychological theory
Source: Implement Sci. 2007 Mar 26;2:8. doi: 10.1186/1748-5908-2-8 (PMC1864990; doi:10.1186/1748-5908-2-8)
Supplement: Additional file 1 — Interview schedule. The questions asked in the interview. [file 1748-5908-2-8-S1.doc]

**Additional file 1. Interview schedule**

1. Have you heard about the new schizophrenia guidelines produced by NICE?

If yes – Are you aware of the recommendations regarding family intervention/therapy?

If yes – What is your understanding of the recommendation for family therapy?

If no – The recommendations are……………….. [KNOWLEDGE]

1. Pre-amble: To what extent do you think the recommendations are being implemented? Can you give me a recent example of it happening? Do you know how to offer family therapy? Do you think that other members of your team know how to offer family therapy? [SKILLS]
2. What are your views about guidelines in general? Does that opinion apply to this guideline? Do you think it is an appropriate part of your job to be following this recommendation? Would following this recommendation create a problem for your professional autonomy? [SOCIAL/PROFESSIONAL ROLE AND IDENTITY]
3. Is it easy or difficult to do? What problems have you encountered? What would help you to overcome these problems? [BELIEFS ABOUT CAPABILITIES]
4. What are the consequences of offering family therapy (prompt for advantages and disadvantages, e.g., time, people, etc.)? Would you say that the benefits outweigh the costs? What would happen if you didn’t offer it? [BELIEFS ABOUT CONSEQUENCES]
5. Do you feel motivated to offer family therapy? Do you feel that you should be offering family therapy? Does offering family therapy conflict with any of your other goals as a health professional? [MOTIVATION AND GOALS]
6. How often do you offer family therapy? What are your reasons for not offering family therapy (prompt for attention, forgetting, time constraints, etc.) [MEMORY, ATTENTION AND DECISION PROCESSES]
7. To what extent do resources influence whether you offer family therapy (prompt for existence of trained staff, time constraints, etc.)? [ENVIRONMENTAL CONTEXT AND RESOURCES]
8. What do you think the views of the other team members are? Do these views influence whether you offer family therapy? [SOCIAL INFLUENCES]
9. Do you think that any emotional factors influence whether family therapy is offered? And what about for you? [EMOTION]
10. Are there procedures or ways of working that encourage offering family therapy? If you see a patient and decide they should be offered family therapy, what are your next steps? [ACTION PLANNING]
